# Analysis of Transcriptomic Changes in Bovine Endometrial Stromal Cells Treated With Lipopolysaccharide

**DOI:** 10.3389/fvets.2020.575865

**Published:** 2020-11-26

**Authors:** Xuefen Ding, Haimiao Lv, Lixin Deng, Wenju Hu, Zhan Peng, Chenbo Yan, Dexin Yang, Chao Tong, Xinzhuang Wang

**Affiliations:** ^1^College of Animal Science and Veterinary Medicine, Henan Agricultural University, Zhengzhou, China; ^2^College of Agricultural Medicine, Henan Radio and Television University, Zhengzhou, China; ^3^Wuhu Overseas Students Pioneer Park, WuHu, China

**Keywords:** whole-genome sequencing, endometritis, bovine, endometria stromal cells, lipopolysaccharide

## Abstract

Endometritis adversely affects the ability of cattle to reproduce and significantly reduces milk production. The is mainly composed of epithelial and stromal cells, and they produce the first immune response to invading pathogens. However, most of the epithelial cells are disrupted, and stromal cells are exposed to an inflammatory environment when endometritis occurs, especially postpartum. Many bacteria and toxins start attacking stromal cell due to loss of epithelium, which stimulates Toll-like receptor (TLRs) on stromal cells and causes upregulated expression of cytokines. Understanding the genome-wide characterization of bovine endometritis will be beneficial for prevention and treatment of endometritis. In this study, whole-transcriptomic gene changes in bovine endometrial stromal cells (BESCs) treated with LPS were compared with those treated with PBS (control group) and were analyzed by RNA sequencing. Compared with the control group, a total of 366 differentially expressed genes (DEGs) were identified in the LPS-induced group (234 upregulated and 132 downregulated genes), with an adjusted *P* < 0.05 by DESeq. Gene Ontology (GO) enrichment analysis revealed that DEGs were most enriched in interleukin-1 receptor binding, regulation of cell activation, and lymphocyte-activated interleukin-12 production. Kyoto Encyclopedia of Genes and Genomes (KEGG) pathway analysis revealed DEGs were most enriched in the TNF signaling pathway, Toll-like receptor signaling pathway, cytokine–cytokine receptor interaction, NF-κB signaling pathway, and chemokine signaling pathway. The results of this study unraveled BESCs affected with LPS transcriptome profile alterations, which may have a significant effect on treatment inflammation by comprehending molecular mechanisms and authenticating unique genes related to endometritis.

## Introduction

Endometritis severely affects the ability of cattle to reproduce, compromises animal welfare, and significantly reduces milk production (1–3). Bacterial infection is the most prevalent element of bovine endometritis, especially, *Escherichia coli*. Lipopolysaccharide (LPS) is a vital constituent of the outer membrane of gram-negative bacteria which can mimic characteristics of an actual gram-negative bacterial infection (4).

Bovine endometrium is mainly composed of bovine endometrial epithelial cells (BEECs) and bovine endometrial stromal cells (BESCs), and they produce the first immune response to invading pathogens (5). Many bacteria and toxins start attacking BESCs due to loss of BEEC after parturition (6–9). Endometritis appears to persist when bacteria of *Trueperella pyogenes* accumulate to a certain amount (9, 10). Characteristics of endometritis caused by *T. pyogenes* are similar to those of pathogenic *E. coli* resulting in bovine endometrium (11). In addition, a previous study showed also that LPS treated bovine endometrial explants *in vitro*, which resulted in luminal epithelial cell shedding and damage. Many bacteria and toxins start attacking BESCs because of losing epithelium, which stimulates Toll-like receptors (TLRs) on BESCs and causes upregulated expression of cytokines (12). BESCs have a more positive role in resisting pyolysin-mediated cytolysis compared with BEECs and are more accessible to the vascular system and mononuclear cells; thus, the effect of stromal cytokines is more significant (3, 13–15). Moreover, stromal cells outnumber epithelial cells and secrete soluble growth factors as much as epithelial cells in order to increase immunological function of uterus endometrium (7, 14–16). So stromal cells play a significant role in initiating bovine endometritis response.

There are lots of studies about BESCs. Researchers have evaluated the effects of LPS and pro-inflammatory mediators (IL-1β and TNF-α) on BEEC and BESC lines by detecting gene expression and the production of cytokine and eicosanoid biosynthesis pathway. Result showed that BEEC and BEEC lines excellently simulate the intrauterine environment (17, 18). In addition, in order to investigate cellular pathways which play essential roles in the regulation of inflammatory response within the endometrial tissue, some studies chose primary isolated BESCs and BEECs and *ex vivo* organ culture (EVOCs) with biological traits. Results showed that influencing the mevalonate pathway is responsible for natural immunity in the endometrium (19). Another study explored the role of TLR3 and retinoic acid-inducible gene I (*RIG-I*) in the innate immune response to bovine herpesvirus-4 (BoHV-4) and ovine viral diarrhea virus (BVDV) and viral pathogen-associated molecular patterns (PAMPs). Results showed that BESCs react against the aggressor of viable viruses by way of in?ammatory cytokine expression, and endometrial cells could check and respond to virus, and their PAMPs, through TLR3 and *RIG-I* (20).

Genome-wide characterization is crucial for understanding the development and pathophysiology of bovine endometritis, which will be beneficial for prevention and treatment of endometritis. Previous studies on RNA-seq analysis in BESC-integrated BEECs treated with LPS showed 20 differentially expressed miRNAs and 108 differentially expressed genes (DEGS) and enriched 118 Gene Ontology (GO) and 66 Kyoto Encyclopedia of Genes and Genomes (KEGG) pathways, which provided an understanding on the effect of miRNA on bovine endometritis (21). Another study on RNA-seq analysis of the change in miRNA and mRNA expressions in 15 cows at 7 and 21 days postpartum showed 4,197 DEGs in healthy cows, while only 31 DEGs in cows with cytological endometritis from pro-inflammatory to hyperplasia stages (22). Other studies have been performed on RNA-seq analysis of the effects of LPS on the whole transcriptome of BEECs with a focus on genes involved in embryo–maternal interactions. They identified 2,035 and 2,073 DEGs in control cells and cells incubated with LPS (23). However, comprehensive transcriptomic analysis of the response of BESCs to LPS by RNA-seq remains to be elucidated.

In this study, genome-wide gene expression profiles in primary BESCs incubated with LPS were evaluated using RNA-seq, and the results were approved by real-time quantitative reverse-transcriptase polymerase chain reaction (qRT-PCR). Under these contents, duties and pathways participating in bovine endometritis, especially postpartum in the BESCs, were analyzed. The logical bioinformatics thinking affirmed that some expressed genes in our study have essential duties in bovine endometritis, especially postpartum, thus proving the reliability of our data.

## Methods

### Bovine Endometrial Stromal Cell Isolation and Primary Culture

Fresh and healthy Holstein bovine with symmetric uterine horns next to the ovary in the pro-estrus time period were acquired from a vicinity abattoir. Bovine health was objectively affirmed by veterinary surgeon examination of the adjudicator from government and individual records of bovine well-being. There is no purulent or mucopurulent vaginal emission or clues of clinical or subclinical endometritis. In addition, uteri were obtained without indication of genital illness or microbial contamination and no ovarian cysts.

Whole stromal cells were isolated from the bovine endometrium as described before with some changes (24, 25). All experimental processes were supported by the Institutional Animal Use Committee of Henan Agricultural University (approval number 2005-0026). Bovine uteri, stored on ice, were moved to the laboratory from a local abattoir. The endometrium was cut into strips and placed in serum-free DMEM/F12 (Gibco, Grand Island, NY, USA) containing 50 IU/ml penicillin, 50 μg/ml streptomycin, and 2.5 μg/ml amphotericin B. Then, endometrial strips were cut into about 1-mm^3^ fragments and placed in phosphate-buffered saline (PBS). They were then digested with 30 ml sterile digestive solution, composed of 60 mg trypsin III (Roche, Lewes, UK), 60 mg collagenase II (Sigma, Poole, UK), and 12 μl deoxyribonuclease I (DNase I, Sigma) in 120 ml PBS at 37°C for about 1 h. The cell suspension was filtered with a 40-μm mesh, and the filtrate was resuspended with 2 ml DMEM/F12 containing 10% fetal bovine serum (FBS, Gibco, Grand Island, NY, USA). After washing the cells five times with DMEM/F12 including 10% FBS, the filtrate was centrifuged at 100 rpm for 10 min at room temperature (RT) (11). The cells were resuspended in DMEM/F12 including 15% FBS, 100 U/ml penicillin, 100 U/ml streptomycin, and 100 U/ml amphotericin B. Cells were seeded in 25-cm^2^ culture flask at a density of 2 × 10^5^ cells/ml and were incubated at 37°C with 5% CO_2_. To obtain stromal cells and remove epithelial cells, cell suspension was transferred 18 h after plating, which resulted to BESCs having a priority attachment opportunity. The medium was changed every 2 days. Cells were passaged with 0.25% trypsin–EDTA when the cells reached ~80% confluence. Cell morphology was recorded and photographed under an inverted microscope.

### Cells Treatment With LPS

BESCs from passage 6 (P6) were planted in a 25-cm^2^ culture flask at a spatial arrangement of 2 × 10^5^ cells/ml. The cells were incubated at 37°C with 5% CO_2_. Once the cells reached 80% confluence, the medium was updated, and cells were cleaned with PBS. We divided the cells into two groups of three duplicates each. DMEM/F12 including 15% FBS and PBS (control) was added to one group, and DMEM/F12 containing 15% FBS and 0.5 μg/ml ultrapure LPS obtained from a pathogenic *E. coli* strain (serotype 055:B5, Sigma-Aldrich, Madison, USA, L2880) was added to the other group and incubated for 12 h each. The LPS concentration of 0.5 μg/ml showed that animals were subclinically infected in the uterine lumen (25, 26).

### RNA Extraction, Quantification, and Qualification

Whole RNA from stromal cells was obtained by RNAisio Plus (Takara, Dalian, Liaoning, China). The concentration and quality of RNA were estimated with a NanoDrop spectrophotometer (Thermo Scientific), and the integrity of RNA was verified with 1% agarose gel. RNA quality was determined using an Agilent Bioanalyzer 2100 (Agilent Technologies, Inc., Santa Clara, CA, USA).

### Library Preparation and Transcriptome Sequencing

Input material of 3 μg of RNA was used for RNA sample preparation. Sequencing libraries were generated using the TruSeq RNA Library Preparation Kit v2 (Illumina, San Diego, CA, USA). In summary, poly-A including mRNA was enriched by oligo-dT magnetic beads, and fragmentation was completed by divalent cations under elevated temperatures in an Illumina proprietary fragmentation buffer. Random oligonucleotides and SuperScript II were used for synthesizing first-strand cDNAs. Second-strand cDNAs were synthesized. Remaining overhangs were converted into blunt ends by exonuclease/polymerase. Before hybridization, 3′ ends of the DNA fragments were adenylated, and Illumina paired-end (PE) adapter oligonucleotides were ligated. The amplified library fragments were enriched using an Illumina PCR primer cocktail in a 15-cycle PCR; then the library size was selected at 300–400 bp fragments. The AMPure XP system was used to purify PCR products, and the Bioanalyzer 2100 system (Agilent, Santa, Clara, CA, USA) was used to detect library size. Finally, library sequencing of PEs was accomplished with next-generation sequencing (NGS) on an Illumina HiSeq platform.

### Quality Control

The raw data of FASTQ were generated by the software of the Illumina HiSeq sequencing platform. Raw data of each sample were counted separately, including sample names, percentage of ambiguous base, Q20, and Q30 sequencing data along with some connectors and low-quality reads. These sequences may cause interference to subsequent data analysis, so sequencing data need to be further filtered. The criteria for data filtering included the following: (1) Cutadapt is used to remove 3′ end connectors, and the removed parts have an overlap of at least 10 bp with known connectors, allowing 20% base mismatch; and (2) reads with an average mass fraction lower than Q20 were removed.

### Mapping Reads to the Reference Genome

The reference genome used was of *Bos taurus*. ARS-UCD1.2.dna.toplevel.fa was downloaded from Ensembl Genome Browser 95. The clean reads were mapped to the reference genome with HISA2 (http://ccb.jhu.edu/software/hisat2/index.shtml).

### Gene Expression Level Quantification

The reads of each gene were counted by HTSeq and normalized by fragments per kilobase of transcript sequence per millions base pairs sequenced (FPKM). FPKM was then calculated (27).

### Analysis of DEGs

Genes that were differentially expressed in two groups (three biological replicates per group) were screened with DESeq. The screening conditions for differentially expressed genes were |log2FoldChange| > 1 and significance of false discovery rate (FDR) < 0.05. FDR is the adjusted *P*-value.

### GO and KEGG Enrichment Analyses of DEGs

We carried out GO enrichment analysis for function annotation and KEGG enrichment analysis for signaling pathway annotation on DEGs (28, 29). GO and KEGG enrichment analyses were completed by the Database for Annotation, Visualization, and Integrated Discovery (DAVID) version 6.7 with threshold of FDR < 0.05 (30).

### qRT-PCR

The genes for qPCR used to confirm the results by NGS were randomly selected (31).

The expression profiles of 10 unselected DEGs were confirmed using SYBR Green-based qRT-PCR using sequence-specific primers (Supplementary Table 1) designed using an online primer design tool (http://frodo.wi.mit.edu/primer3/). The cDNA samples were compounded by reverse transcription of coequal amounts of total RNA from stromal cells treated with LPS (experimental group) and PBS (control group), with three duplicates each, using the cDNA synthesis kit (Takara, Dalian, Liaoning, China) according to the manufacturer's manual. The reverse transcription was first accomplished in a 10-μl reaction volume to remove the genomic DNA reaction containing RNA samples, 1 μl; 5×gDNA Eraser Buffer, 2 μl; gDNA Eraser, 1 μl; and dd H_2_O, 6 μl. Reaction conditions included 42°C for 2 min and 4°C for 1 min, which followed a 20-μl reaction volume reaction containing a 10-μl reaction volume of the previous step: 1 μl Prime Script RT Enzyme Mix, 1 μl Prime Mix, 4 μl 5×Prime Script Buffer, and 4 μl dd H_2_O. Reaction conditions included 37°C for 15 min, 85°C for 5 s, and 4°C 1 min. Primer specificity was tested by first performing a conventional PCR and confirmed by the presence of a single melting curve during qRT-PCR. Serial dilutions (1:10, 1:20, 1:50, and 1:200) were made from a pool of cDNA, and calibration curves were performed for each gene. Afterwards, qRT-PCR was then performed in a 20-μl reaction volume containing TB Green Premix Ex Taq II, 10 μl (Takara, Dalian, Liaoning, China); cDNA samples, 1 μl; specific forward and reverse primers, 1 μl (respectively); and dd H_2_O, 7 μl in 0.1-ml white PCR eight-strip tubes (NEST Biotechnology, Wuxi, China) with the CFX96 real-time PCR detection system (Bio-Rad, Munich, Germany). Reaction conditions included pre-degeneration at 95°C for 30 s, followed by 40 cycles of degeneration at 95°C for 5 s and annealing at 60°C for 30 s. After finishing the process of each PCR, the dissociation curves served as an affirmation of the specificity of the amplification. The abundance of each transcript in each sample was determined using a comparative threshold cycle Ct (2^−ΔΔ^Ct) method as expounded previously (32). The data of qRT-PCR were analyzed after the Ct value of the target genes was normalized with the Ct value of glyceraldehyde-3-phosphate dehydrogenase (GAPDH). qRT-PCR was carried out in three biological replicates. Student's *t*-test or the least significant difference test procedure was engaged to demonstrate mRNA expression differences between the samples. Differences with *P* < 0.05 were considered significant. Statistical significance of the data was demonstrated by Student's *t*-test carried out with SPSS (PASW Statistics for Windows, Version 18.0, Chicago: SPSS Inc., USA).

## Results

### Immunofluorescence Analysis

Stromal cells exhibited flat, fibroblast-like morphology (Supplementary Figure 1). Endometrial stromal cells were estimated by immunostaining for vimentin (stromal cells marker). Stromal cell expression was vimentin positive and cytokeratin-18 negative (Supplementary Figure 2). Cell morphology and the positive staining for vimentin confirmed that the cultured cells were indeed stromal cells.

### Quality Control of the RNA Sequence

The RIN values of RNA samples including LPS1, LPS2, LPS3, PBS1, PBS2, and PBS3 were 9.8, 10, 9.9, 10, 10, and 9.8, respectively (Supplementary Figure 3). And the results of RNA-specific agarose electrophoresis showed that the three bands were 28s, 18S, and 5S. The brightness of the three bands decreases gradually, which was brightest in 28s and darkest in 5S. The width of the 28s band is twice as wide as that of 18S (Supplementary Figure 4). These indicated that RNA quality was fine. We established three RNA libraries for the control group and three RNA libraries for the LPS-induced group. After filtering the low-quality reads, the average number of clean reads was 42,381,010 (92.67%) and 44,167,270 (92.77%) for the control group and LPS-induced group, respectively (Table 1). The clean reads were used for the following analyses, and most of them (>93.99%) were mapped to the *Bos taurus* genome (Table 2).

**Table 1:** Data filtering statistics after Illumina sequencing.

**Sample**	**Raw reads**	**Clean reads No**.	**Clean data (bp)**	**Clean reads (%)**	**Q20 (%)**	**Q30 (%)**
PBS1	47,469,396	44,046,138	6,650,966,838	92.78	96.08	91.45
PBS2	46,293,936	42,854,784	6,471,072,384	92.57	96.49	92.24
PBS3	43,441,712	40,242,110	6,076,558,610	92.63	96.25	91.79
LPS1	42,929,910	39,806,986	6,010,854,886	92.72	96.19	91.59
LPS2	50,813,700	47,094,016	7,111,196,416	92.67	96.16	91.6
LPS3	49,082,750	45,600,808	6,885,722,008	92.9	96.42	91.9

*N%: The percentage of fuzzy bases. Q20: The percentage of bases with a Phred value >20. Q30: The percentage of bases with a Phred value > 30*.

**Table 2:** Summary of clean reads mapped to the reference genome.

**Sample**	**Clean_reads**	**Total_mapped**	**Multiple_mapped**	**Uniquely_mapped**
PBS1	44,046,138	41,598,276 (94.44%)	2,394,502 (5.76%)	39,203,774 (94.24%)
PBS2	42,854,784	39,556,838 (92.30%)	2,206,194 (5.58%)	37,350,644 (94.42%)
PBS3	40,242,110	37,935,794 (94.27%)	2,240,965 (5.91%)	35,694,829 (94.09%)
LPS1	39,806,986	37,586,818 (94.42%)	2,233,726 (5.94%)	35,353,092 (94.06%)
LPS2	47,094,016	44,530,730 (94.56%)	2,676,831 (6.01%)	41,853,899 (93.99%)
LPS3	45,600,808	43,007,562 (94.31%)	2,461,139 (5.72%)	40,546,423 (94.28%)

*Clean Reads: The total number of sequences used for alignment. Total Mapped: The total number of sequences identical to the reference genome in clean reads; percentage is total mapped/clean reads. Multiple Mapped: The total number of sequences aligned to multiple locations; percentage is multiple mapped/total mapped. Uniquely Mapped: The total number of sequences aligned to only one location; percentage is uniquely mapped/total mapped*.

### Identification of the Source of Variance in the Expressed Transcripts by Principal Component Analysis (PCA)

The purpose of PCA is to account for the origin of variance in our data. We performed PCA with two elements: PC1 and PC2. PC1 was 55%, and PC2 was 42% (Supplementary Figure 5). PCA can cluster similar samples together; the closer the distance, the higher the similarity between samples. This indicated that these data could be used further.

### Differential Gene Expression

**Figure 1:**
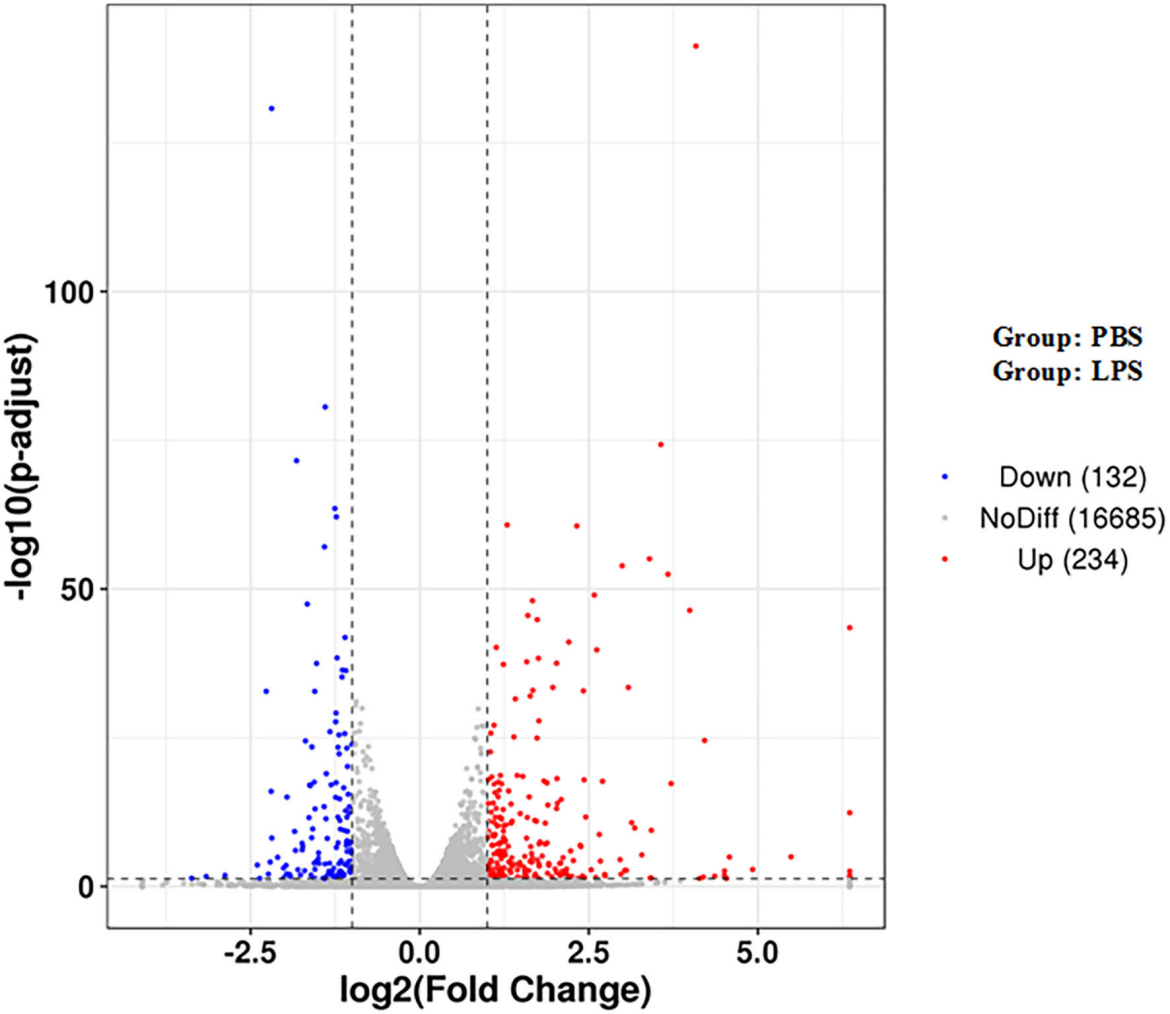
Volcano map of DEGs. The two vertical dotted lines are the threshold of the differential expression. The horizontal dotted line is the threshold FDR at 0.05. Upregulated and downregulated genes are shown as red and blue dots, and gray dots represent non-significantly differentially expressed genes.

There were a total of 366 DEGs (composed of 234 upregulated and 132 downregulated DEGs) identified in the LPS-induced group compared to the control group. Some of these genes are related to inflammation, such as inflammatory cytokines including interleukin-1 alpha (*IL1A*), interleukin-2 receptor β (*IL2RB*), and interleukin-6 (*IL6*), which increased by 3.16-, 20.53-, 15.88-fold, respectively. Cytokines including chemokine (C-C motif) ligand 2 (*CCL2*), *CCL5*, interleukin-8 (*CXCL8*), and *CXCL2* increased by 16.91-, 10.48-, 3.39-, 2.14-fold, respectively. Antimicrobial factor *DEFB10* increased by 2.79-fold (Figure 1 and Supplementary Table 2).

### The Heatmap of DEGs

**Figure 2:**
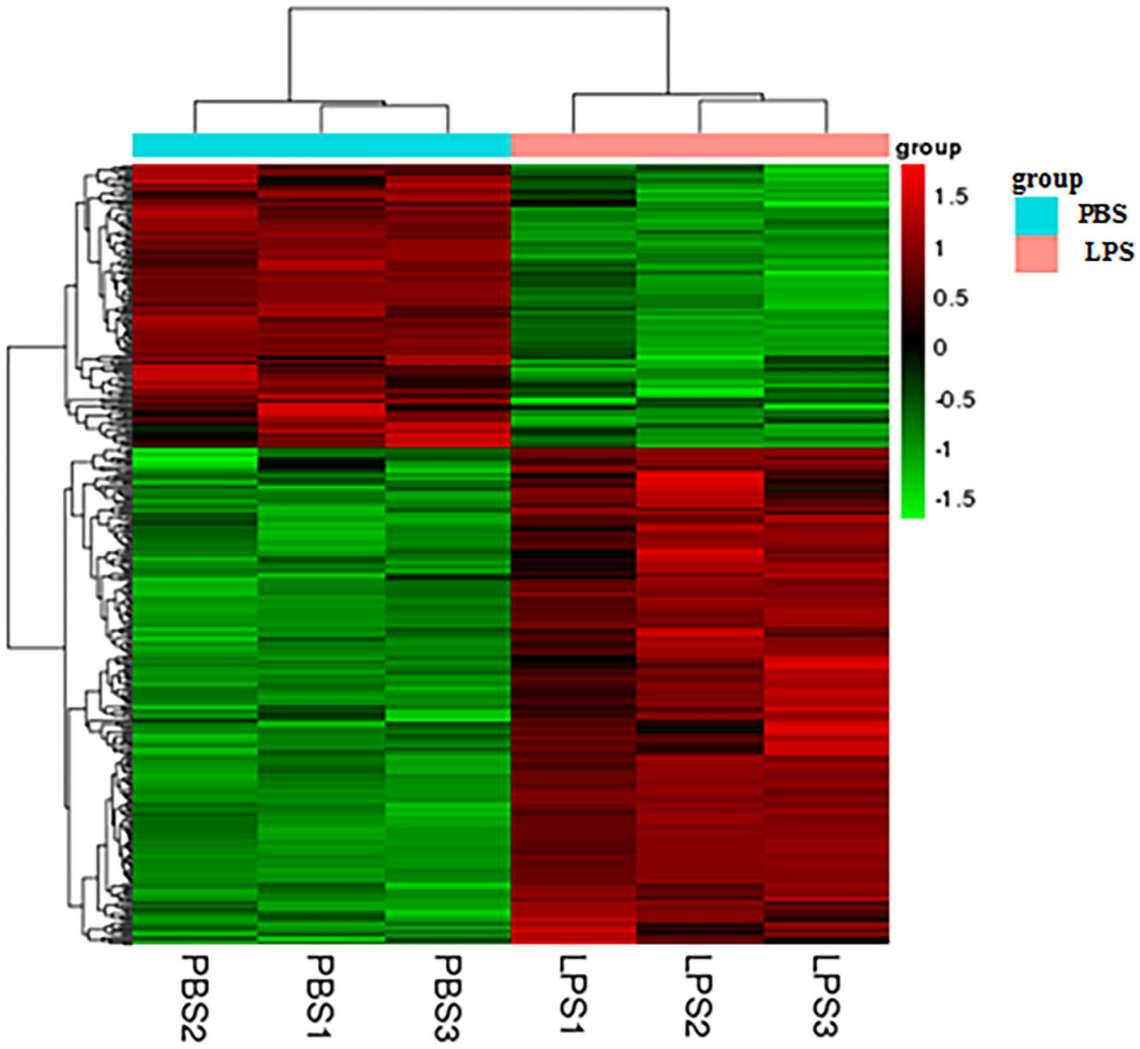
Heatmap analysis of DEGs. The horizontal lines represent genes, and each column is a sample. Red represents high-expression genes, and green represents low-expression genes. The X-axis is the sample number, and the Y-axis is the DEGs.

Heatmap analysis of DEGs identified genes of expression levels with high correlation among samples. Some of these genes may be involved in biological processes, metabolic processes, or signaling pathways (Figure 2).

### GO Enrichment of DEGs

**Figure 3:**
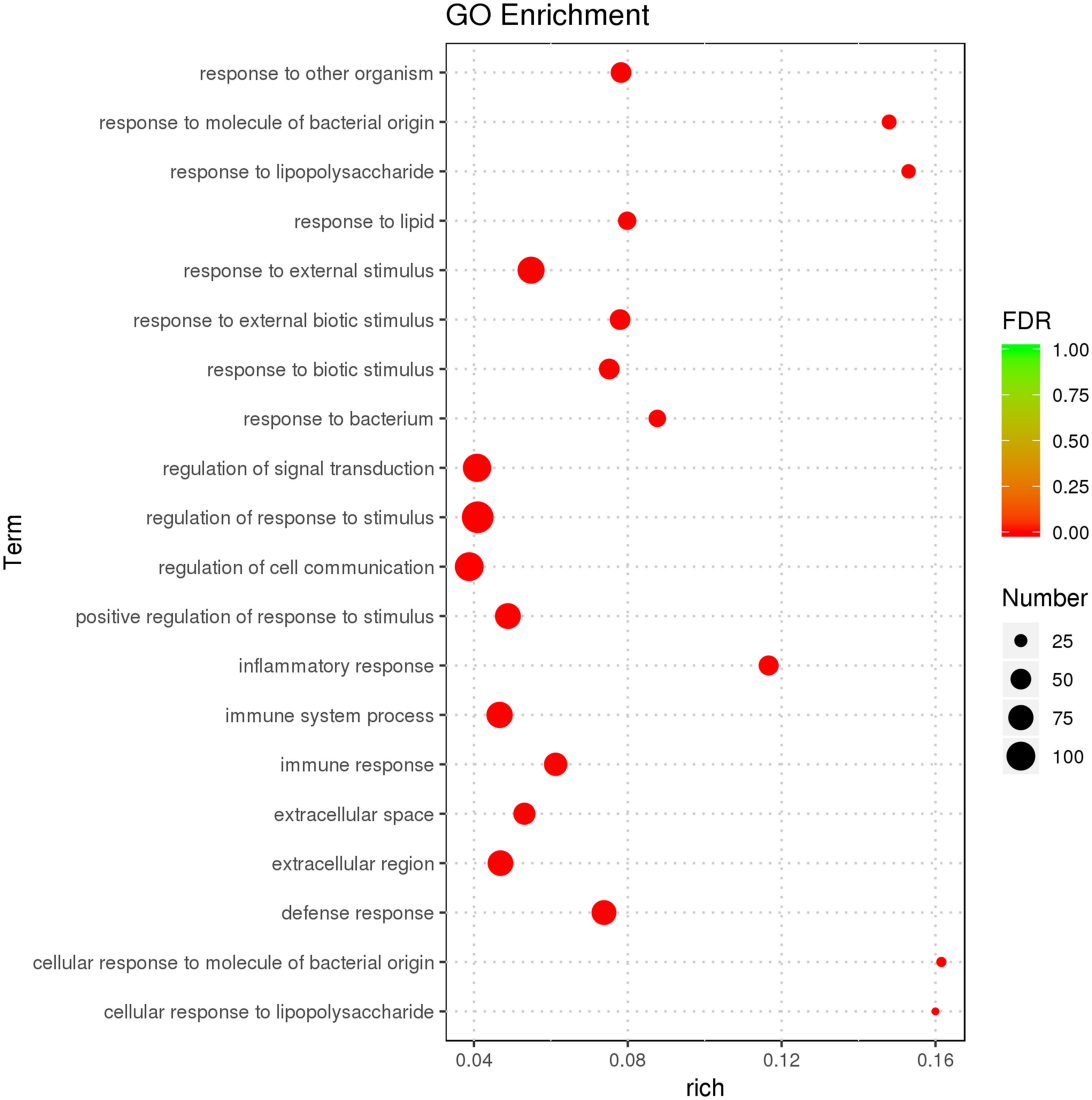
GO enrichment analysis displaying the first 20 GO terms with the most significant enrichment.

GO enrichment was used to characterize DEGs. DEGs were enriched in immunity-related GO terms, such as leukocyte-mediated immunity, intracellular signal transduction, IL-1 receptor binding, IL-1β secretion, and response to cytokines. There were 638 GO terms, comprised by 574 biological processes (BPs), 21 cellular components (CCs), and 43 molecular functions (MFs). GO enrichment results for all DEGs are illustrated (Supplementary Table 3), and the top 20 GO terms with the most significant enrichment were selected (Figure 3).

### KEGG Enrichment Analysis

**Figure 4:**
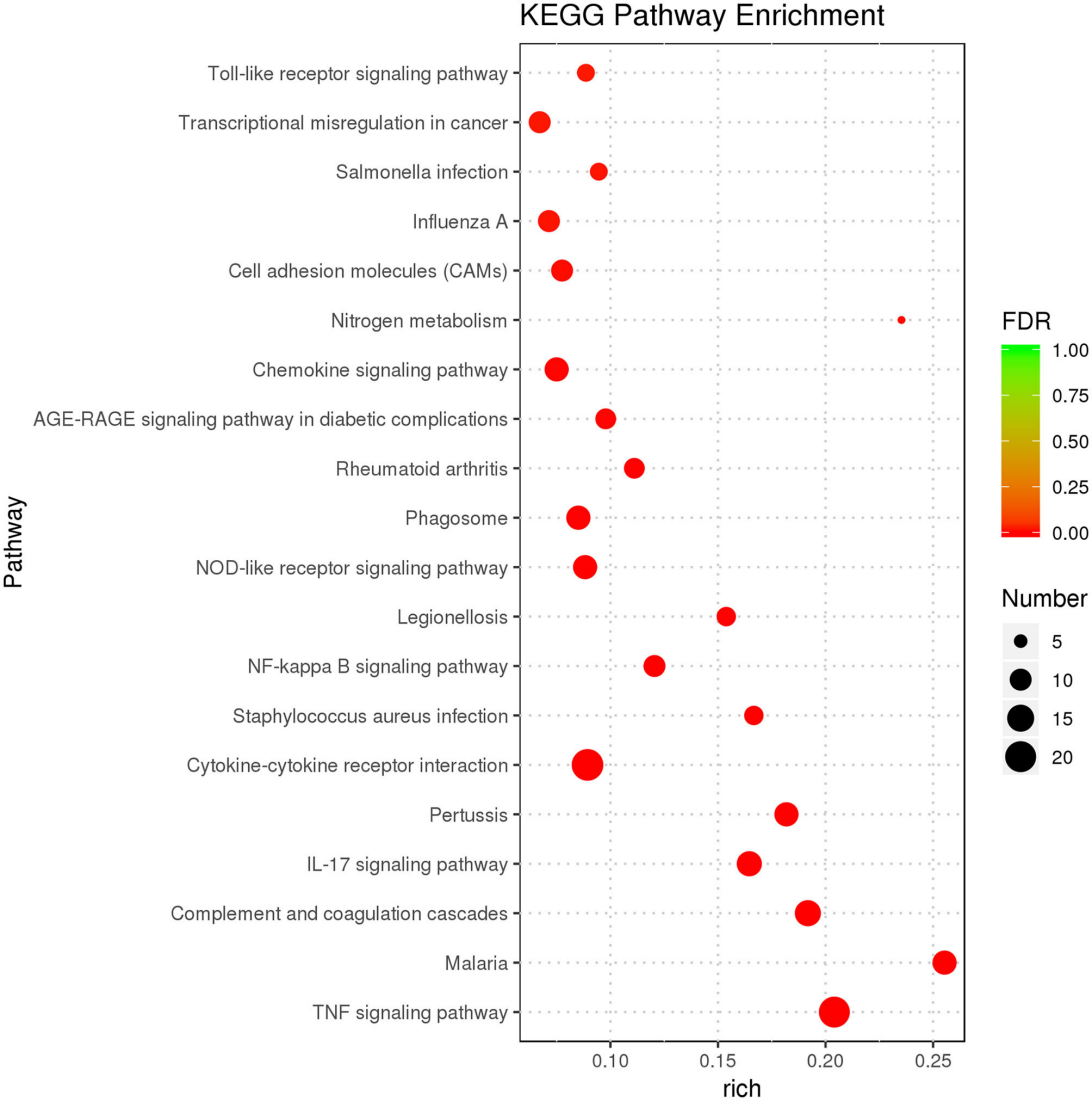
KEGG enrichment analysis displaying the first 20 KEGG terms with the most significant enrichment.

The purpose of KEGG enrichment analysis is to appraise pathways that are vital in inflammation in endometrial stromal cells. The results of the analysis showed that inflammation pathways in endometrial stromal cells are mainly centered on the TNF signaling pathway, complement and coagulation cascades, cytokine–cytokine receptor interaction, IL-17 signaling pathway, chemokine signaling pathway, nucleotide-binding oligomerization domain-like (NOD-like) receptor signaling pathway, NF-κB signaling pathway, and so on. KEGG enrichment results of DEGs are illustrated (Supplementary Table 4). The top 20 KEGG terms with the most significant enrichment were selected (Figure 4).

### Confirmation of DEGs by qRT-PCR

**Figure 5:**
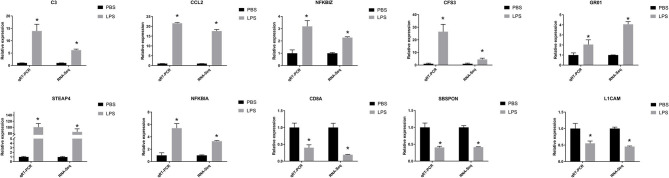
QRT-PCR verified features of DEGs by RNA-seq. The relative expression level of target mRNAs was calculated using the 2^−ΔΔ*Ct*^ method and expressed relative to the value in the control group. Results were displayed in mean ± SEM (*n* = 3). Log_2_ fold change was the ratio of average log_2_ folds between groups. *P < 0.05.

We selected 10 DEGs which included seven upregulated genes, *NFKBIZ, NFKBIA, CCL2*, complement component 3 (*C3*), colony-stimulating factor 3 (*CSF3*), growth-regulated oncogene 1 (*GRO1*), and prostate 4 (*STEAP4*), and three downregulated genes, L1 cell adhesion molecule (*L1CAM*), T-cell marker (*CD8A*), and *SBSPON*, and confirmed them by real-time qRT-PCR (as shown in Figure 5). The expression levels of the 10 DEGs were found to be similar with RNA-seq results.

## Discussion

**Figure 6:**
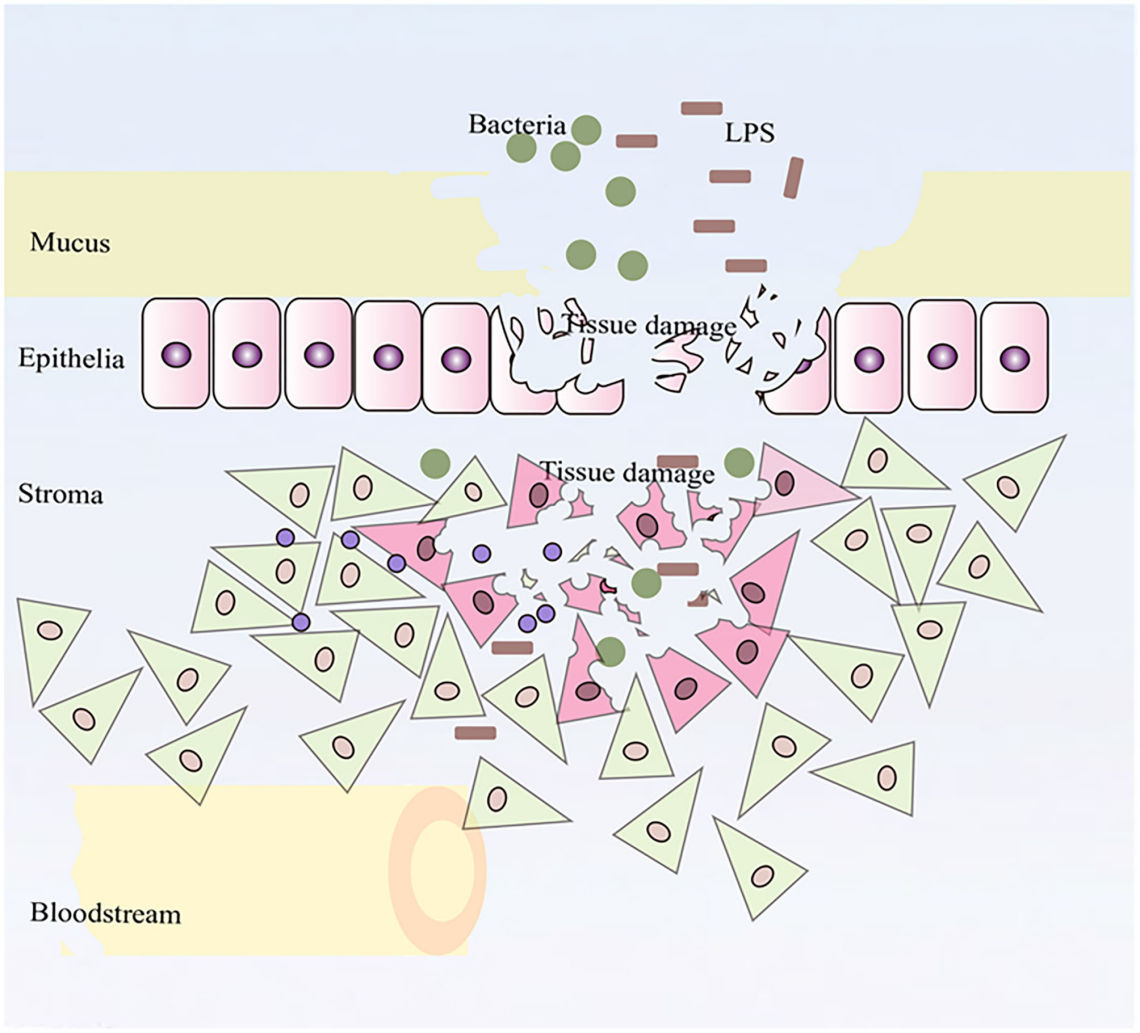
Stromal cells were exposed to an inflammatory environment when epithelial cells were disrupted.

**Figure 7:**
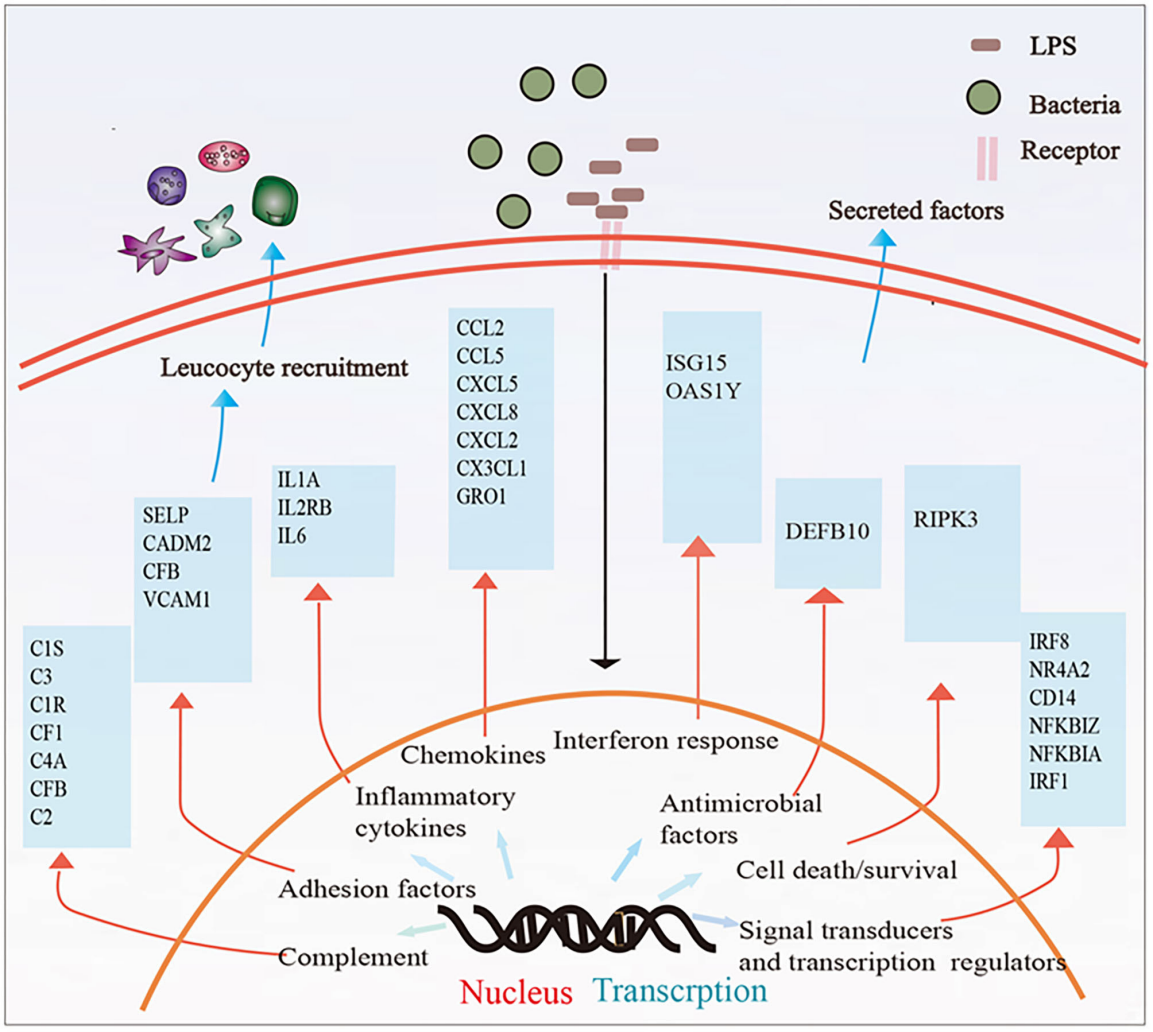
Summary of the immune response in BESCs exposed to bacterial LPS. LPS treatment for 12 h changed the mRNA expression of many genes involved in inflammatory and innate immune response.

In this study, we described whole transcriptomic gene changes in BESC stimulated with LPS compared with the control group by RNA-seq. Stromal cells are exposed to an inflammatory environment when epithelial cells are disrupted (Figure 6); inflammation on BESC is activated (Figure 7).

We chose P6 BESC. A previous study used P5 BEEC to analyze effects of LPS on the whole transcriptome with an uncommon concern on genes related to embryo–maternal interactions (23). And transcriptome profiling investigation of interactions between P6 bovine vaginal epithelial cell and an isolated lactobacillus strain was performed (33). Another study used P6 beard dermal papilla cells to study interactions between transiently transfected androgen receptors and Hic-5/ARA55 (34). Another work has supported the expression of mRNA, and some pro-inflammatory factors are not affected by cell passaging in human gingival fibroblasts (35). However, some studies indicated some differences in mRNA expression between P0 and P3 in primary bovine oviductal epithelial cells (BOEC) *in vitro*, such as the transcription rate of MUC4 and encoded proteins of IL8. But culture passaging has no effect in mRNA expression of some genes, such as *OXCT2*, prostaglandin E2 (*PGE2*), cyclooxygenase-2 (*PTGS2*), and polypeptide chemokine *IL8* mRNA (36). Likewise, BOEC treated with *T. pyogenes* showed not only different mRNA expressions of some genes but also similar mRNA expressions in P0 and P3 compared with the controls (without *T. pyogenes*) (37). Cells in this study of P1 to P3 were pure, but the numbers were not enough. So we think the P6 cell expression profile is not exactly the same as P1, but P6 cells still represent most of the characteristics of primary stromal cells.

The main function of the serum is to provide basic nutrients in *in vitro* cell culture. We use 15% serum instead of 10% in order to provide enough nutrition for cells to grow better, such as culturing primary bovine vaginal epithelial cells and bovine endometrial luminal epithelial cells *in vitro* with 15% FBS (33, 38).

LPS binds to the lipoprotein binding protein (LBP), which presents it to the CD14 receptor. This induces CD14 to present the LPS–LBP complex to myeloid differentiation factor 2 (MD-2) (39, 40). This, in turn, promotes dimerization of Toll-like receptor 4 (TLR4)/MD-2, activating two downstream signaling pathways including the MyD88-dependent pathway (MyD88) and TRIF-dependent pathway. The former triggers NF-κB and mitogen-activated protein kinase (MAP kinase) signaling and induces inflammatory cytokines, and the latter causes the induction of type I interferons (IFNs) through IFN regulatory factor 3 (IRF3) activation and inflammatory cytokines through NF-κB activation (39). Our data present evidence for the activation of the MyD88-independent pathway as LPS upregulated inflammatory cytokines, such as *IL6, IL1A*, and *CXCL8* and activated the NF-κB pathway. Bovine endometrial epithelial and stromal cells express the TLR4/CD14/MD2 receptor complex. We found increased expression levels of CD14 but constant levels of TLR4 or MyD88 in our study, consistent with previous studies that exposed mixed BEECs and BESCs to LPS (41). In addition, LPS upregulated TLR4 only in BEECs incubated with 1 μg/ml LPS for 6 and 12 h (42).

After the membrane surface receptor is recognized, intracellular inflammation is recognized, and intracellular signaling cascades are activated by LPS, which is mediated by innate pattern recognition receptors (PRRs) including TLR, retinoic acid-inducible gene-I-like (RIG-I-like) receptors, NOD-like receptors, and C-type lectin receptors (43). These lead to the expression of inflammatory mediators and contribute to the clearance of pathogens (44). Our data further confirm these findings because LPS triggers the NOD-like receptor, TLR, and C-type lectin receptor signaling pathway. Previous studies show that mRNA of proinflammatory cytokines interleukin-1 alpha (*IL1A*), *IL1B, IL6*, and *TNF* and expression of chemokines *IL8* and *CXCL5* increased in endometrial epithelial cells during the estrous cycle and subclinical or clinical endometritis (45, 46). Similarly, in the present study, we observed an increase in many immune-related DEGs, such as *IL6, IL1A, IL2RB, CCL2, CCL5*, granulocyte chemotactic protein 2 (*CXCL5*), *CXCL8, CXCL2*, CX3C, chemokine ligand 1 (*CX3CL1*), and *CCL20*. We suggest these genes may serve as a sign for the discovery of bovines with subclinical endometritis and for supervising new therapy approaches and may assist in the development of more targeted and effective vaccines and drugs for the prevention and treatment of bovine endometritis. In addition, stromal cells play positive roles in resisting viable viruses by causing expression of inflammatory cytokines and chemokines (20). The antiviral defense system of BESCs demands integrated recognition of PAMPs, originally via TLR3 and later via stimulating RIG-I (20). Therefore, we suggest that stromal cells also are vital immunoregulatory cells.

Local immune responses are activated, which results in expression of pro-inflammatory cytokines when the uterus is exposed to bacteria. This is followed by production of antimicrobial peptides (AMPs) and acute phase proteins by epithelial and innate immune cells (3). Many AMP genes are expressed, including lingual antimicrobial peptide (*LAP*), tracheal antimicrobial peptide (*TAP*), and some β-defensins (3, 45, 47). Some studies have identified an increase in expression levels of TAP, LAP, DEFB1, and DEFB5 in the endometrium of cows with serious inflammation or following LPS treatment *in vitro* (42, 48, 49). Other studies showed that LAP, TAP, and neutrophil β-defensins (*BNBD4* and *DEFB5*) were all upregulated in bovine epithelial cells but not stromal cells with LPS (3, 5). In this study, we found that *DEFB10* was upregulated in bovine stromal cells with LPS.

The cascade reaction of complement signals are also responsible for immune defense, and complement activation leads to opsonization of pathogens and their removal by phagocytes and cell lysis (50). Our results showed that some complement genes were upregulated, such as *C3, C1S, C1R*, chloroplast F1 (*CF1*), and *C4A*. LPS can change the expression level of many genes involved in cell adhesion, such as vascular cell adhesion molecule-1 (*VCAM-1*), silk-elastin-like protein polymers (*SELP*), cell adhesion molecule 2 (*CADM2*), hepatocyte cell adhesion molecule (*HEPACAM*), and sidekick 2 (*SDK2*). Leukocytes attach to cell adhesion molecules (CAM) on endothelial cells and are involved in inflammation and immune functions (51). Each CAM has an inherent effect on the immune response process, such as VCAM-1, which promotes firm binding of T cells and induces transmigration (52, 53).

Our results also expound on the cellular processes in the endometrium, which paves the way for future elucidation of molecular mechanisms of microbial invasion and host cell response. We analyzed the interactions between LPS and stromal cells including interaction of LPS with surface receptors of stromal cells and intracellular signaling cascades activated by PRRs. These indicate that stromal cells play a key role in bovine endometrium reaction to foreign intruders by initiating or inhibiting biochemical and molecular signals.

We suggest that, in addition to BEEC or other cell types in the endometrium, BESC can be studied further as targets for the treatment of endometritis. A finer statement of the interplay between epithelial cells and stromal cells within the endometrium is important in developing strategies for improved bovine endometritis.

## Conclusions

RNA-seq showed many important immune-related genes and signaling pathways in BESCs in the LPS-induced group as compared to the control group, which may have participated in the pathological process of bovine endometritis, especially postpartum, and deserves further study and discussion. Our analysis paves the way for the future elucidation of the molecular mechanisms of microbial invasion and host cell response.

## Data Availability Statement

The sequence data of this study have been deposited into Sequence Read Archive (http://www.ncbi.nlm.nih.gov/sra, accession number PRJNA574911). The datasets supporting the conclusions of this article are included in this article and its supplementary information files.

## Ethics Statement

The animal study was reviewed and approved by Institutional Animal Use Committee of Henan Agricultural University (approval number. 2005-0026) and Beijing Association for Science and Technology [approval SYXK (Beijing) 2007-0023].

## Author Contributions

XW, CT, and XD designed the study and revised the manuscript. XD, HL, LD, WH, ZP, CY, and DY performed the study. XD, HL, LD, CT, and XW analyzed the data. XD, HL, and LD wrote the manuscript. All authors read and approved the final manuscript. All authors contributed to the article and approved the submitted version.

## Conflict of Interest

The authors declare that the research was conducted in the absence of any commercial or financial relationships that could be construed as a potential conflict of interest.

## Supplementary Material

Supplementary Figure 1 Morphological characteristics of P6 BESC. BESC were fibroblast-like and enlarged at the time of confluence after which they overgrew in multiple layers. (Scale bars =100um).

Supplementary Figure 2 Immunofluorescence staining shows positive expression of vimentin expression (green) and negative expression of cytokeratin-18 in BESC (Scale bars =50um).

Supplementary Figure 3 The RIN values of RNA including LPS 1, LPS 2, LPS 3, PBS 1, PBS 2, PBS 3.

Supplementary Figure 4 RNA quality image. M: DNA Marker; 1: PBS 1; 2: PBS 2; 3: PBS 3; 4: LPS 1; 5: LPS 2; 6: LPS 3.

Supplementary Figure 5 PCA analysis of the expressed transcripts. The X-axis is the first principal component, and the Y-axis is the second principal component. Different shapes represent different samples, and different colors represent different groups.

Supplementary Table 1 QRT-PCR primers used in the study.

Supplementary Table 2 DEGs in the BESC were treated with LPS compared with PBS (control). The upregulated genes are shown in purple; the downregulated genes are shown in blue. Inf: infinite.

Supplementary Table 3 The significant GO terms of DEGs.

Supplementary Table 4 KEGG enrichment analysis of DEGs.

## Abbreviations

BESC: bovine endometrial stromal cells
BEEC: bovine endometrial epithelial cells
LPS: lipopolysaccharide
DEGs: differentially expressed genes
GO: Gene Ontology
KEGG: Kyoto Encyclopedia of Genes and Genomes
PPARs: peroxisome proliferator-activated receptors
RNA-Seq: high-throughput mRNA sequencing
PCA: principal component analysis
LBP: lipoprotein binding protein
PRRs: innate pattern recognition receptors
AMP: antimicrobial peptides
LAP: lingual antimicrobial peptide
TAP: tracheal antimicrobial peptide
MD-2: myeloid differentiation factor 2
FDR: false discovery rate
IRF3: interferon regulatory factor 3
MAP kinase: mitogen-activated protein kinase
ISG: IFN-stimulated genes
GAPDH: glyceraldehyde 3-phosphate-dehydrogenase
STEAP4: six-transmembrane epithelial antigen of prostate 4
CAM: cell adhesion molecules
VCAM-1: vascular cell adhesion molecule-1
HA: hyaluronan
ECM: extracellular matrix
HAS2: hyaluronan synthase 2.
