# Supplementary figures and images for: Analysis of Transcriptomic Changes in Bovine Endometrial Stromal Cells Treated With Lipopolysaccharide
Source: Front Vet Sci. 2020 Nov 26;7:575865. doi: 10.3389/fvets.2020.575865 (PMC7725876; doi:10.3389/fvets.2020.575865)

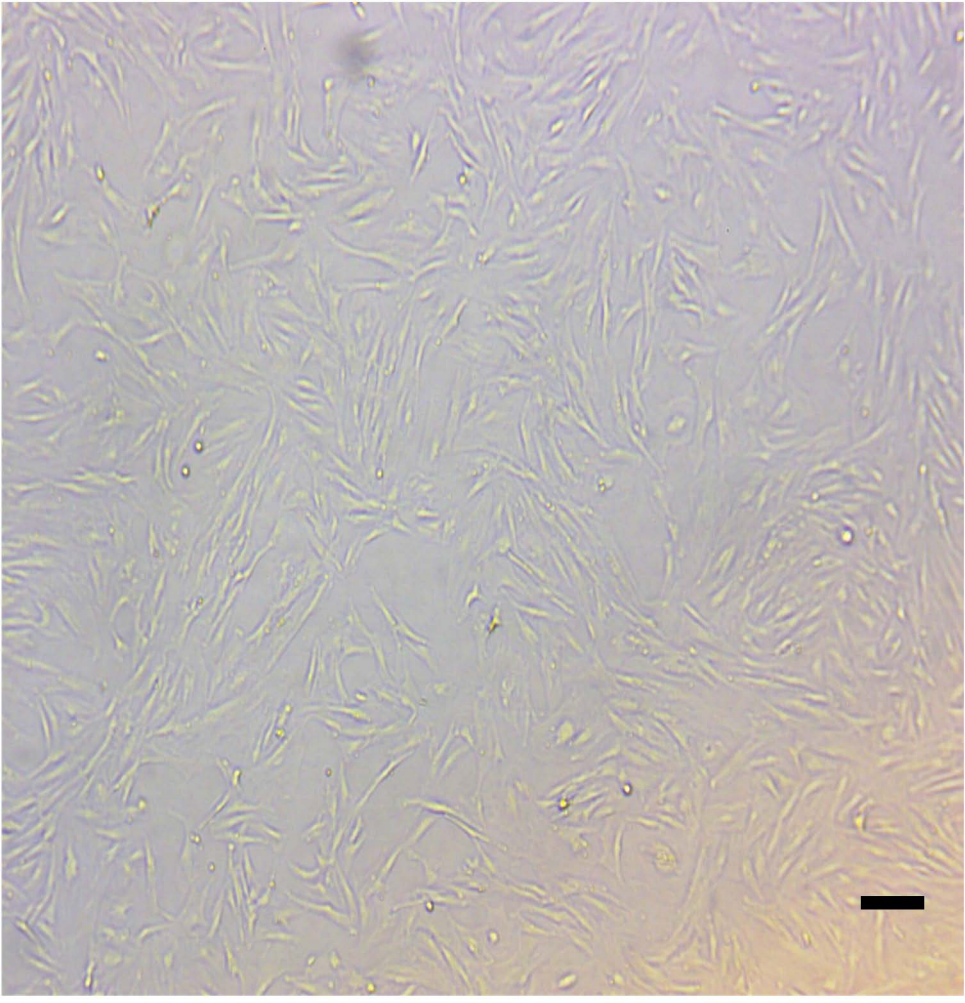

Supplement: Supplementary Figure 1 — Morphological characteristics of P6 BESC. BESC were fibroblast-like and enlarged at the time of confluence after which they overgrew in multiple layers. (Scale bars =100um). [file Image_1.pdf]

DL2,000 DNA Marker

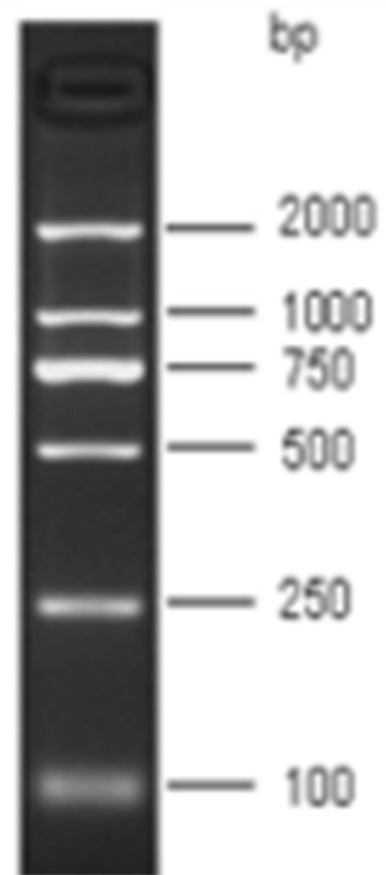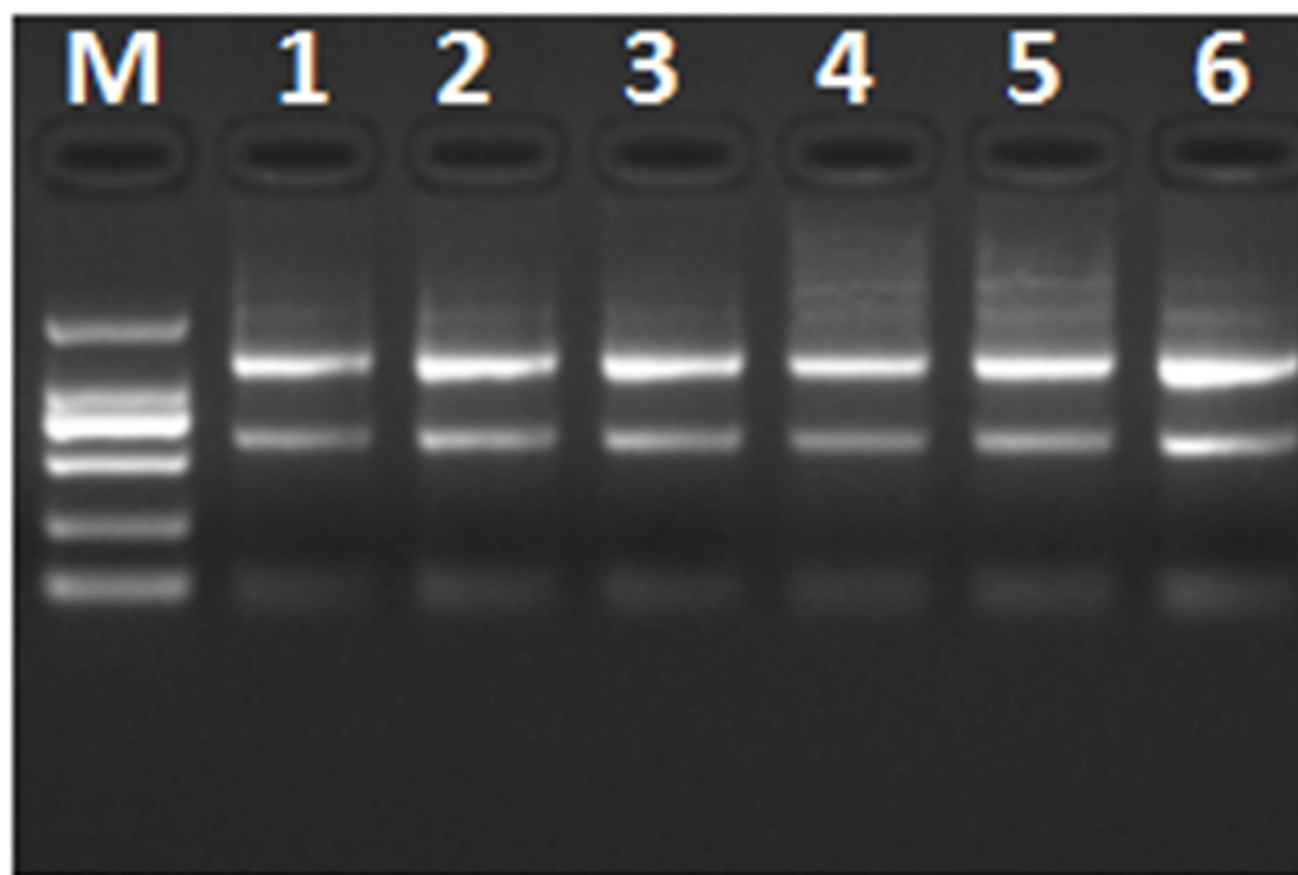

Supplement: Supplementary Figure 4 — RNA quality image. M: DNA Marker; 1: PBS 1; 2: PBS 2; 3: PBS 3; 4: LPS 1; 5: LPS 2; 6: LPS 3. [file Image_4.pdf]

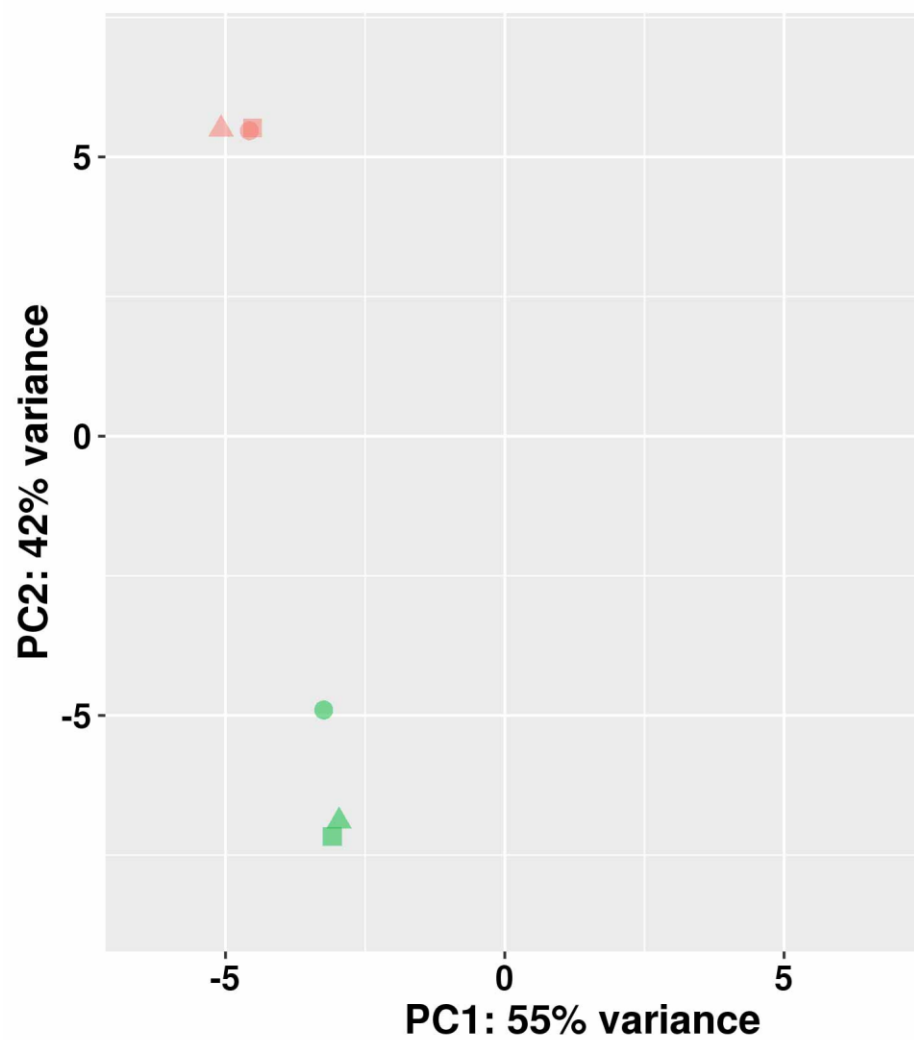

Replication

- rep 1
- rep 2
- rep 3

Condition

- PBS
- LPS

Supplement: Supplementary Figure 5 — PCA analysis of the expressed transcripts. The X-axis is the first principal component, and the Y-axis is the second principal component. Different shapes represent different samples, and different colors represent different groups. [file Image_5.pdf]
