# Supplementary material for: Analysis of Transcriptomic Changes in Bovine Endometrial Stromal Cells Treated With Lipopolysaccharide
Source: Front Vet Sci. 2020 Nov 26;7:575865. doi: 10.3389/fvets.2020.575865 (PMC7725876; doi:10.3389/fvets.2020.575865)
Supplement: Supplementary Figure 3 — The RIN values of RNA including LPS 1, LPS 2, LPS 3, PBS 1, PBS 2, PBS 3. [file Image_3.pdf]

LPS1

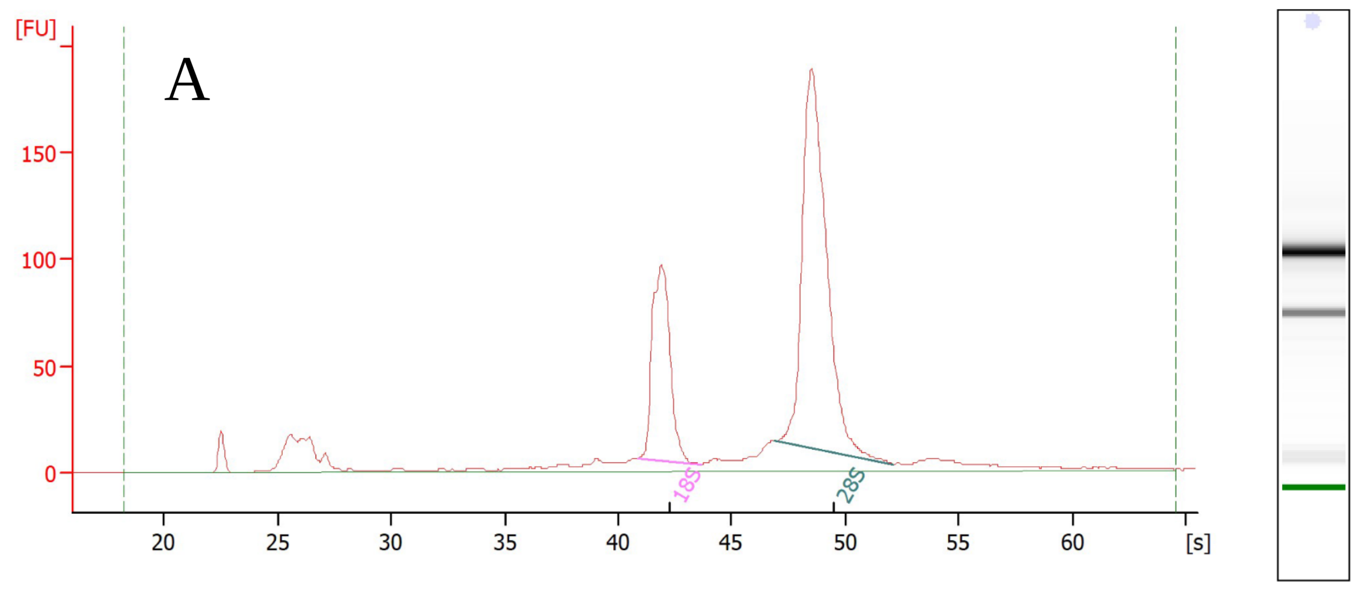

Overall Results for sample 11 : LPS1

RNA Area:	1,042.4	RNA Integrity Number (RIN):	9.8 (B.02.09)
RNA Concentration:	361 ng/µl	Result Flagging Color:	
rRNA Ratio [28s / 18s]:	2.2	Result Flagging Label:	RIN: 9.80

Fragment table for sample 11 : LPS1

Name	Start Time [s]	End Time [s]	Area	% of total Area
18S	40.84	43.64	201.4	19.3
28S	46.92	52.11	447.3	42.9

LPS2

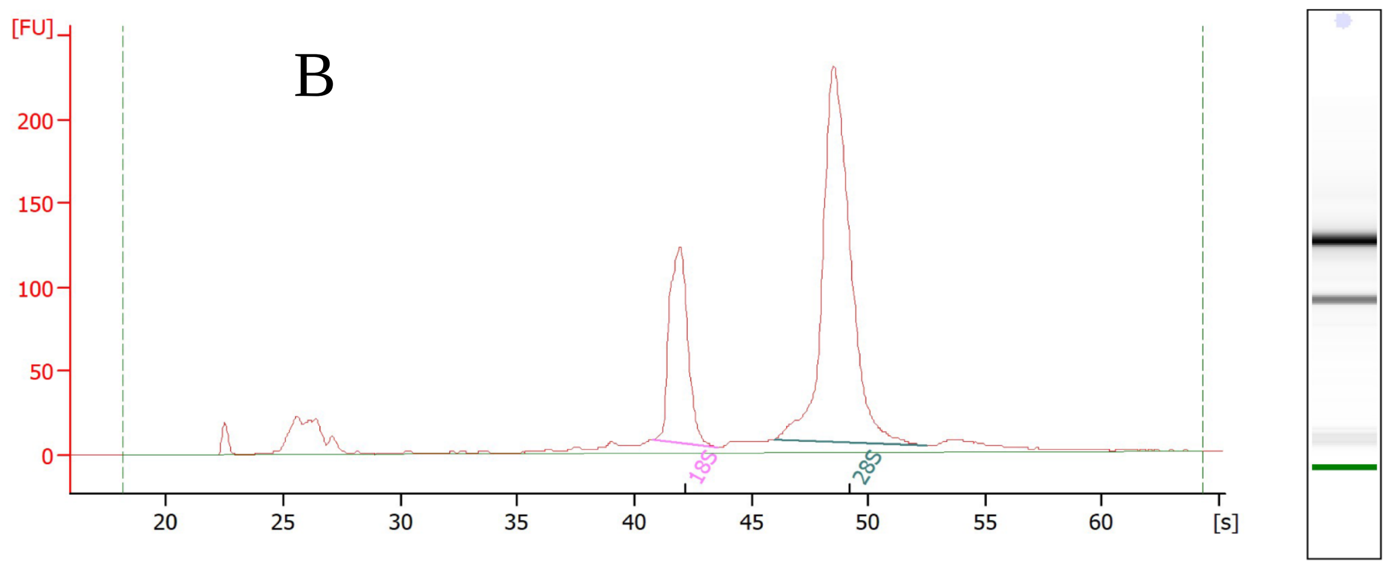

Overall Results for sample 12 : LPS2

RNA Area:	1,298.7	RNA Integrity Number (RIN):	10 (B.02.09)
RNA Concentration:	450 ng/µl	Result Flagging Color:	
rRNA Ratio [28s / 18s]:	2.5	Result Flagging Label:	RIN:10

Fragment table for sample 12 : LPS2

Name	Start Time [s]	End Time [s]	Area	% of total Area
18S	40.76	43.60	256.6	19.8
28S	45.98	52.50	634.7	48.9

LPS3

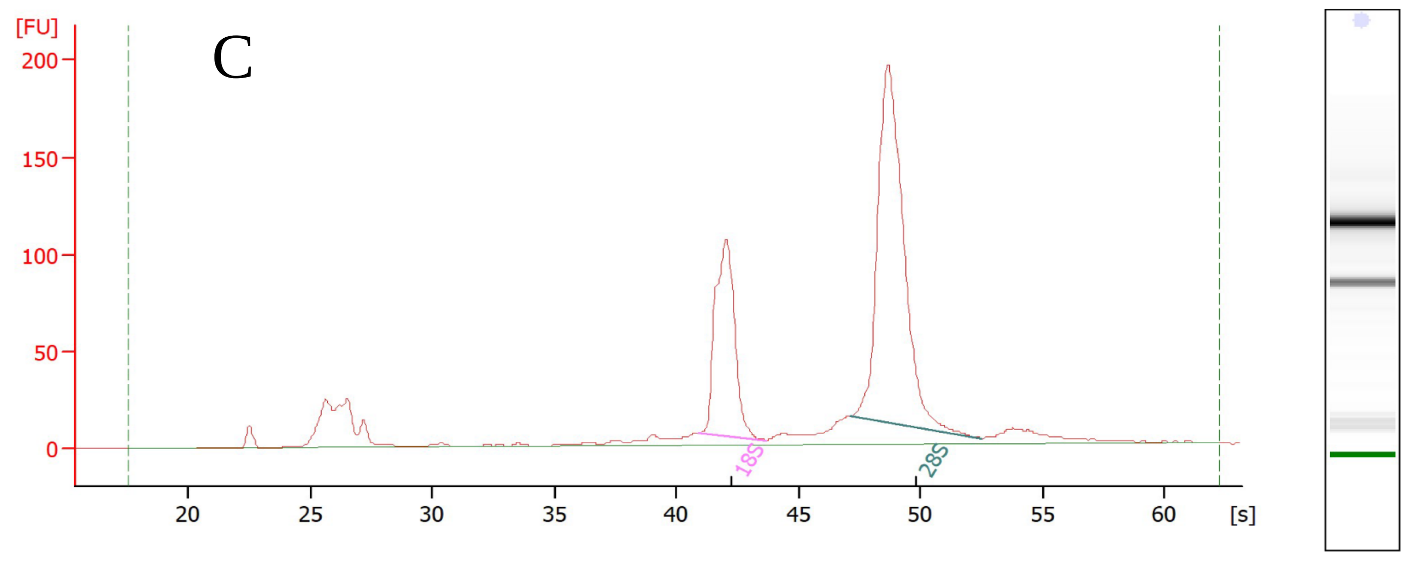

Overall Results for sample 12 : LPS3

RNA Area:	1,127.1	RNA Integrity Number (RIN):	9.9 (B.02.09)
RNA Concentration:	534 ng/µl	Result Flagging Color:	
rRNA Ratio [28s / 18s]:	2.1	Result Flagging Label:	RIN: 9.90

Fragment table for sample 12 : LPS3

Name	Start Time [s]	End Time [s]	Area	% of total Area
18S	40.94	43.65	221.8	19.7
28S	47.12	52.48	468.1	41.5

PBS1

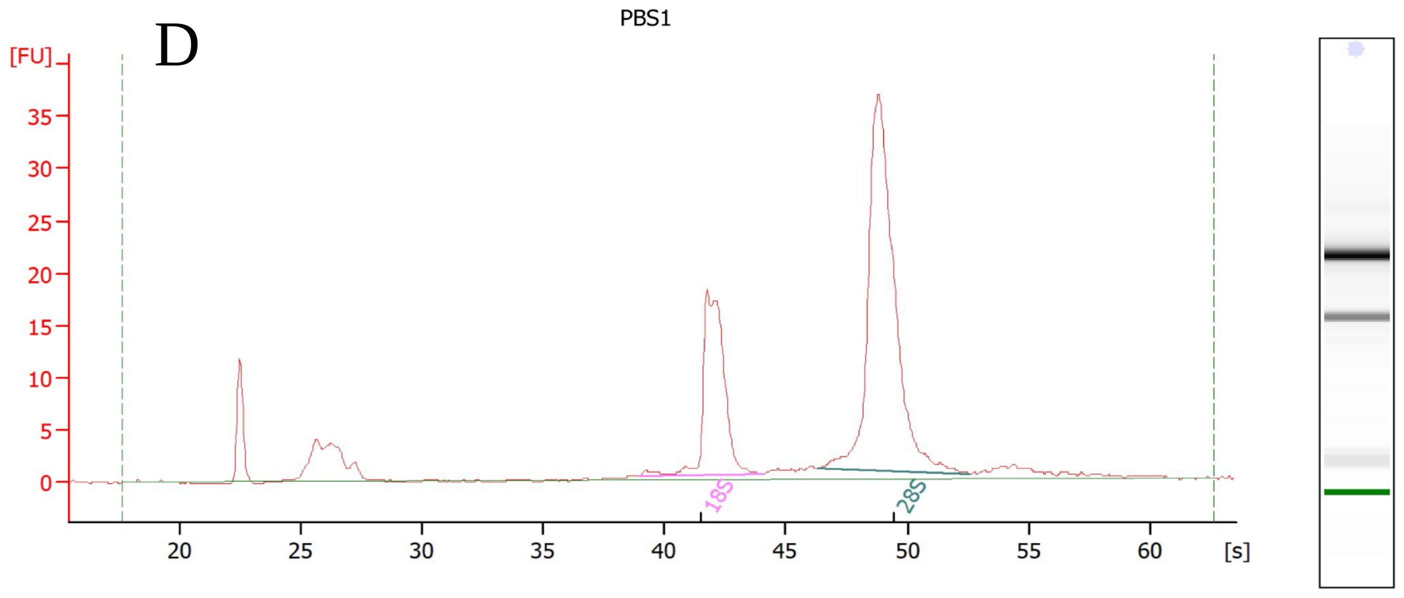

Overall Results for sample 9 : PBS1

RNA Area:	193.8	RNA Integrity Number (RIN):	10 (B.02.09)
RNA Concentration:	92 ng/µl	Result Flagging Color:	
rRNA Ratio [28s / 18s]:	2.2	Result Flagging Label:	RIN:10

Fragment table for sample 9 : PBS1

Name	Start Time [s]	End Time [s]	Area	% of total Area
18S	38.92	44.00	42.4	21.9
28S	46.32	52.58	92.6	47.8

PBS2

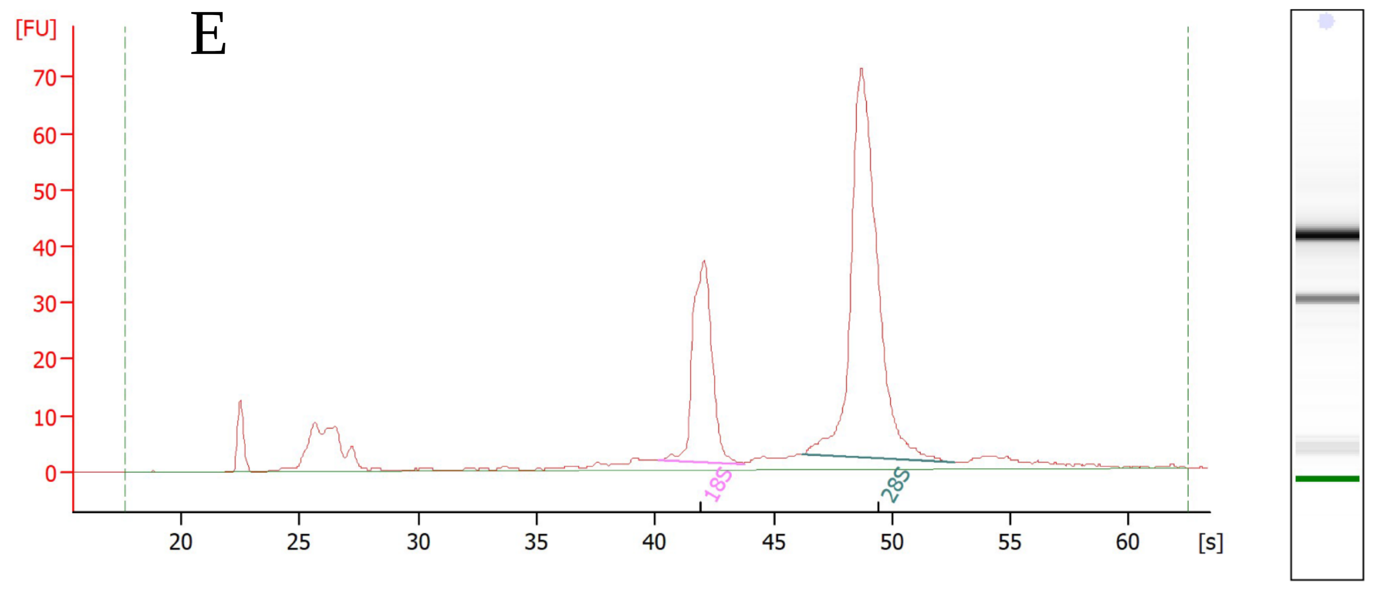

Overall Results for sample 10 : PBS2

RNA Area:	394.9	RNA Integrity Number (RIN):	10 (B.02.09)
RNA Concentration:	187 ng/µl	Result Flagging Color:	
rRNA Ratio [28s / 18s]:	2.3	Result Flagging Label:	RIN:10

Fragment table for sample 10 : PBS2

Name	Start Time [s]	End Time [s]	Area	% of total Area
18S	40.16	43.73	80.1	20.3
28S	46.22	52.65	183.0	46.4

PBS3

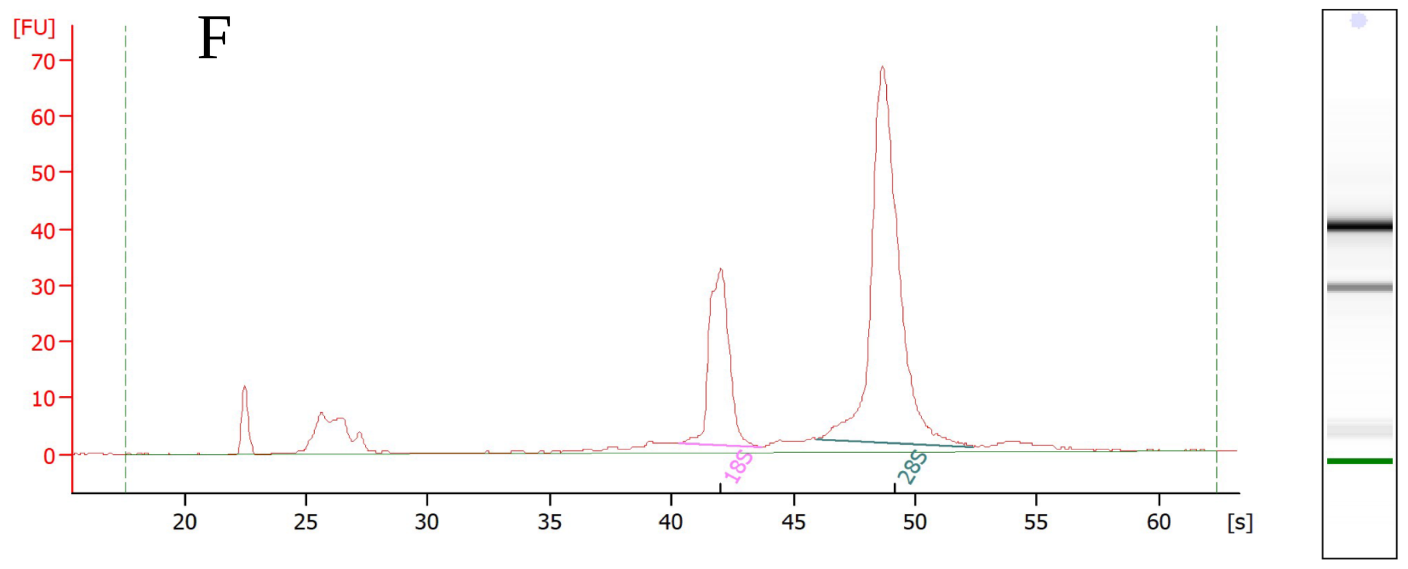

Overall Results for sample 11 : PBS3

RNA Area:	367.6	RNA Integrity Number (RIN):	9.8 (B.02.09)
RNA Concentration:	174 ng/µl	Result Flagging Color:	
rRNA Ratio [28s / 18s]:	2.5	Result Flagging Label:	RIN: 9.80

Fragment table for sample 11 : PBS3

Name	Start Time [s]	End Time [s]	Area	% of total Area
18S	40.35	43.69	70.6	19.2
28S	45.95	52.36	176.1	47.9
