# Supplementary material for: Analysis of Transcriptomic Changes in Bovine Endometrial Stromal Cells Treated With Lipopolysaccharide
Source: Front Vet Sci. 2020 Nov 26;7:575865. doi: 10.3389/fvets.2020.575865 (PMC7725876; doi:10.3389/fvets.2020.575865)
Supplement: Supplementary Table 1 — QRT-PCR primers used in the study. [file Table_1.doc]

| **Table 1_v1. QRT-PCR primers used in the study** | | | |  |
| --- | --- | --- | --- | --- |
| Gene | GenBank accession no. | Forward primer 5’-3’ | Reverse primer 5’-3’ | Product size |
| *L1CAM* | NM_001192435.1 | GCCTTATGTCCACTATACCTTTCG | GCATTCCAGTCCATCCACC | 196 |
| *NFKBIZ* | NM_174726.1 | TCGGGTGACGCAGTTGG | GACTTTCCCTTCAGGATACG | 147 |
| *NFKBIA* | NM_001045868.1 | CGAGGAGTATGAGCAGATGGT | AGCGATTTCTGGCTGGTTAG | 265 |
| *CCL2* | NM_174006.2 | CCTCCTGTGCCTGCTAC | TTGCTGCTGGTGACTCTT | 156 |
| *C3* | NM_001040469.2 | ACGCCGCCTTCCAAT | TGTGCCTCGCAAATGTCT | 286 |
| *STEAP4* | XM_002686859.5 | CCACTAACTCCTGGCTCA | TCTTCCCACCGTACACC | 198 |
| *CSF3* | NM_174028.1 | GTCACTGACTTTGCCACGAACA | AGCGCAGGCCACGGTAT | 181 |
| *GRO1* | NM_175700.2 | AACCGAAGTCATAGCCAGTCTCA | GGCACTAGCCTTGTTTAGCATCT | 106 |
| *SBSPON* | NM_001075869.2 | TTGTGCCAGCCAGTGTCG | CCTTGTTGAACGCAGAGGTAGTT | 191 |
| *CD8A* | NM_174015.1 | CGGCGTCCTTCTCCTGTCA | TGGGCTTGCCTCCTTGTCG | 107 |
| *β-actin* | NM_173979.3 | AACTCCATCATGAAGTGTGACG | GATCCACATCTGCTGGAAGG | 234 |
