# Supplementary material for: Analysis of Transcriptomic Changes in Bovine Endometrial Stromal Cells Treated With Lipopolysaccharide
Source: Front Vet Sci. 2020 Nov 26;7:575865. doi: 10.3389/fvets.2020.575865 (PMC7725876; doi:10.3389/fvets.2020.575865)
Supplement: Supplementary Figure 2 — Immunofluorescence staining shows positive expression of vimentin expression (green) and negative expression of cytokeratin-18 in BESC (Scale bars =50um). [file Image_2.pdf]

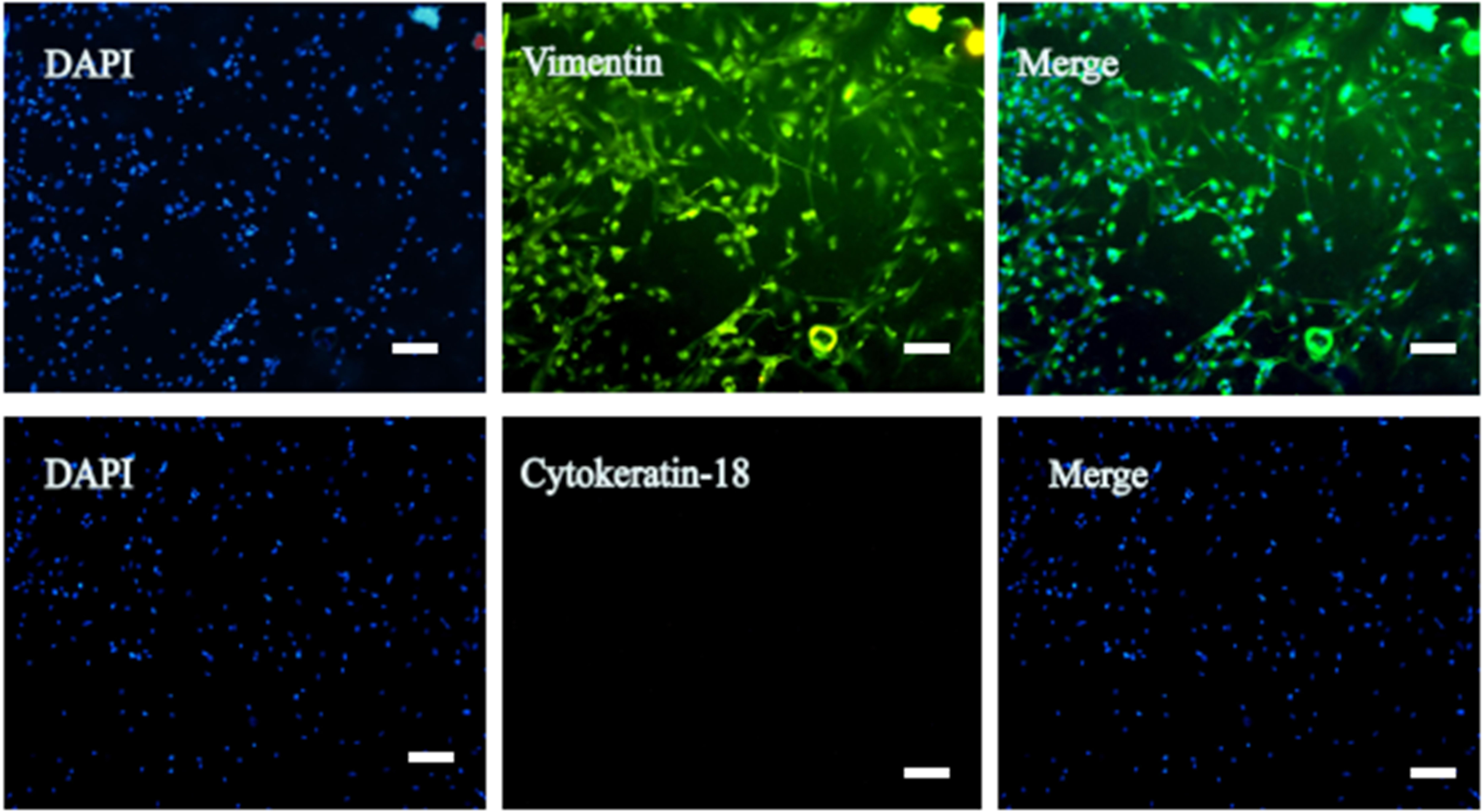
DAPI
Vimentin
Merge
DAPI
Cytokeratin-18
Merge
